# *Ab initio* molecular dynamic study of solid-state transitions of ammonium nitrate

**DOI:** 10.1038/srep18918

**Published:** 2016-01-12

**Authors:** Hongyu Yu, Defang Duan, Hanyu Liu, Ting Yang, Fubo Tian, Kuo Bao, Da Li, Zhonglong Zhao, Bingbing Liu, Tian Cui

**Affiliations:** 1 State key Laboratory of Superhard Materials, College of Physics, Jilin University, Changchun, 130012, P. R. China; 2 Department of Physics and Engineering Physics, University of Saskatchewan, Saskatoon, S7N 5E2, Canada

## Abstract

High-pressure polymorphism and phase transitions have wide ranging consequences on the basic properties of ammonium nitrate. However, the phase diagram of ammonium nitrate at high pressure and high temperature is still under debate. This study systematically investigates the phase transitions and structural properties of ammonium nitrate at a pressure range of 5–60 GPa and temperature range of 250–400 K by ab initio molecular dynamics simulations. Two new phases are identified: one corresponds to the experimentally observed phase IV’ and the other is named AN-X. Simultaneously, the lattice strains play a significant role in the formation and stabilization of phase IV’, providing a reasonable explanation for experimental observation of phase IV-IV’ transition which only appears under nonhydrostatic pressure. In addition, 12 O atoms neighboring the N_H_ (N atom in ammonium cation) atom are selected as reference system to clearly display the tanglesome rotation of ammonium cation.

Ammonium nitrate (AN, NH_4_NO_3_), which is a common material, is attractive for agricultural use because of its high nitrogen content^1^,^2^, and is also a good insensitive explosive^3^ when mixed with other materials. AN was first synthesized by Johann Rudolf Glauber in 1659, and mass produced during the development of the synthetic ammonia industry in the middle of the twentieth century. In recent decades, AN has been used as an eco-friendly and inexpensive energetic solid rocket propellant material^4^,^5^. Similar to other energetic materials, the basic properties of AN are severely affected by phase transitions and high-pressure polymorphism^6^,^7^. For example, at about 300 K and ambient pressure, the phase IV-III transition involves a significant volume change which restricts the use of AN as an efficient oxidizer^4^. Hence, previous researchers have tried to determine the phase diagram of AN by X-ray diffraction (XRD), neutron diffraction, and Raman spectroscopy measurements.

At normal pressure, AN exists in six phases at temperature range of 0–442 K. The lowest temperature phase VII is discovered in 1954 (below 103 K), which is further confirmed by Theoret and Sandorfy (below 213 K)^8^. Except phase VII, the crystallographic information of other five phases are determined using neutron diffraction: *Pccn* for phase V^9^,^10^ (below 255 K); *Pmmn* for phase IV^11^,^12^,^13^ (255–305 K); *Pnma* for phase III^13^,^14^ (305–357 K); *P*4/2_1_*m* for phase II^12^,^15^ (357–398 K); and *Pm3m* for phase I^14^ (398–442 K). The above five phases of AN are also differed by motional freedom and disorder of ammonium cations and nitrate anions^16^. In phase V, both [NH_4_]^+^ and [NO_3_]^−^ are ordered. In phase IV, both [NH_4_]^+^ and [NO_3_]^−^ retain their orientational order with the [NH_4_]^+^ reorientation frequency. In phase III, the [NH_4_]^+^ are disordered, but the [NO_3_]^−^ are ordered. Both [NH_4_]^+^ and [NO_3_]^−^ in phase II and I are disordered. Both the crystal structure of AN and the behavior of [NH_4_]^+^ are significantly affected by temperature. In addition, phase IV-III transition only occurs in the presence of extremely little water; otherwise phase IV directly transforms into phase II^14^.

Besides the above six phases at atmospheric pressure, there are five phases at high pressures. Phase VI occurs at temperature above 442 K and pressure greater than 0.9 GPa^17^. Phase VIII, which is stable between 0.45 and 2.7 GPa, transforms into phase IX above 2.7 GPa and room temperature.^18^ However, the crystallographic information of phases VI, VIII, and IX are unclear. In 1994, Sandstrom *et al.*^19^ found a shock-induced phase change at 3.5 GPa, but they did not provide the structure information. Recently, Davidson *et al.*^20^ suggested a metastable isostructural transition (IV-IV’) at about 20 GPa under nonhydrostatic compression, but there is no phase transition until 35 GPa under hydrostatic compression. Simultaneously, they elaborated the structure of phase IV’, which is similar to that of phase IV with subtle difference in the hydrogen-bonding network. Subsequently, Dunuwille *et al.*^21^ further affirmed the phase IV-IV’ transition using pressure-induced Raman spectral changes. Sorescu and Thompson studied the isotropic compression of phase IV using ab initio total energy calculations and found that phase IV was stable up to 600 GPa.^22^ More recently, phase IV is considered to be stable up to 45 GPa at room temperature using synchrotron X-ray diffraction (XRD) and Raman spectroscopy measurements.^23^ There are still numerous uncertainties in phase IV-IV’ transition and higher pressure phases of AN.

Theoretical studies can make a detailed understanding of AN at the atomic level, such as melting point, energy bands, density of states, the nature of hydrogen bonding, and the structural and electronic properties.^22^,^24^,^25^,^26^ In this work, we use the supercell (Fig. 1a) of phase IV as initial structure to explore the phase transition of AN from 5 to 60 GPa through ab initio molecular dynamics simulations. The results shows that the phase IV’ is confirmed and a new phase, AN-X, is discovered. In addition, we select 12 O atoms neighboring the N_H_ (N atom in [NH_4_]^+^) as reference system to show the rotation details of [NH_4_]^+^ in different phases.

## Results and Discussion

Before obtaining crystal structure from MD trajectories, it is indispensable to investigate the atomic vibration of a system, which can be done by calculating the atomic mean square displacement (MSD) as a function of time using:


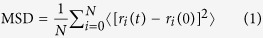


where *N* is the total number of atoms in the system, *r*_*i*_(*t*) is the atomic position at a time of *t* and *r*_*i*_(0) is the initial position of atom *i*, and < … > represents the ensemble average.

The MSD derived from MD simulations at 5 GPa and 300 K are shown in Fig. 2. It can be seen that the MSD of N and O atoms are approaching to a constant, indicating that they only slightly vibrate near the equilibrium position. So, the equilibrium positions of N and O atoms can be obtained by averaging their MD trajectories after the systems reach equilibrium state (about 5 ps). However, the MSD of H atoms can be clearly seen to fluctuate wildly with time, indicating that the above method doesn’t work for hydrogen atoms. Observing the particles’ trajectories over time, it is found that this trouble is caused by the rotation of [NH_4_]^+^. Therefore, another method is adopted to get the equilibrium positions of H atoms. Firstly, H atoms are shifted into the supercell according to periodicity condition at every MD step to obtain the “H atom cloud” (see Fig. 3). Then, the positions of H atoms can be obtained by making statistical average over the “H atom cloud”.

**Figure 1:**
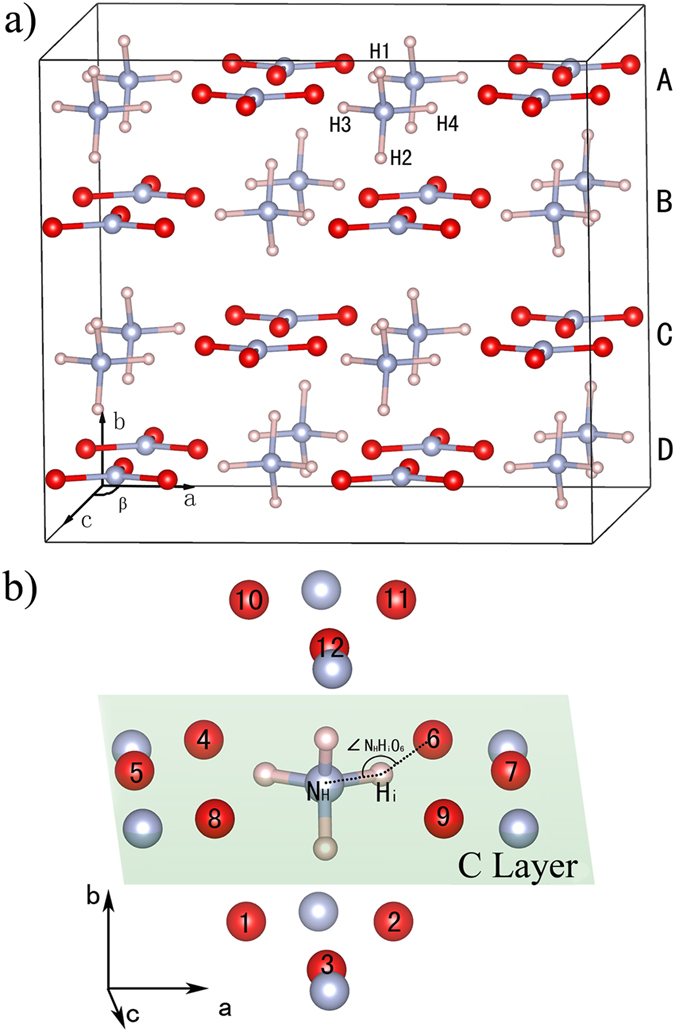
Crystal structure of 2 × 2 × 2 AN-IV supercell and reference system. (**a**) A, B, C and D represent 4 layers of supercell, respectively. (**b**) Reference system for ammonium ion. Atoms O_1_–O_3_ are in D layer, O_4_–O_9_ are in C layer and O_10_–O_12_ are in B layer. Blue, red and pink spheres represent nitrogen, oxygen, and hydrogen, respectively.

**Figure 2:**
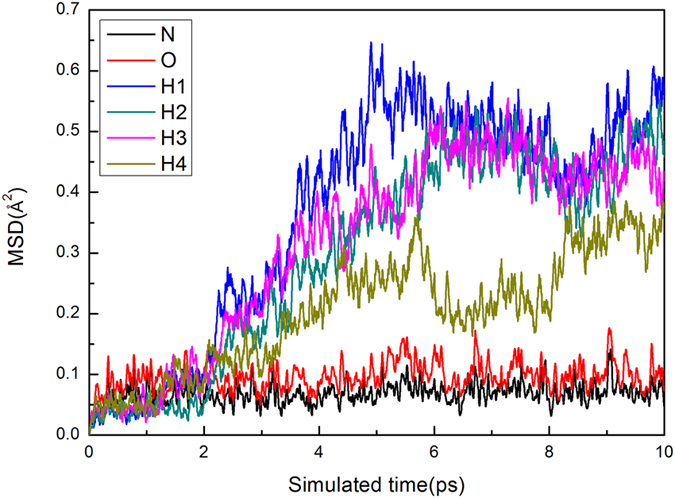
Simulated MSD. Simulated MSD as a function of time at 5 GPa and 300 K for phase IV.

**Figure 3:**
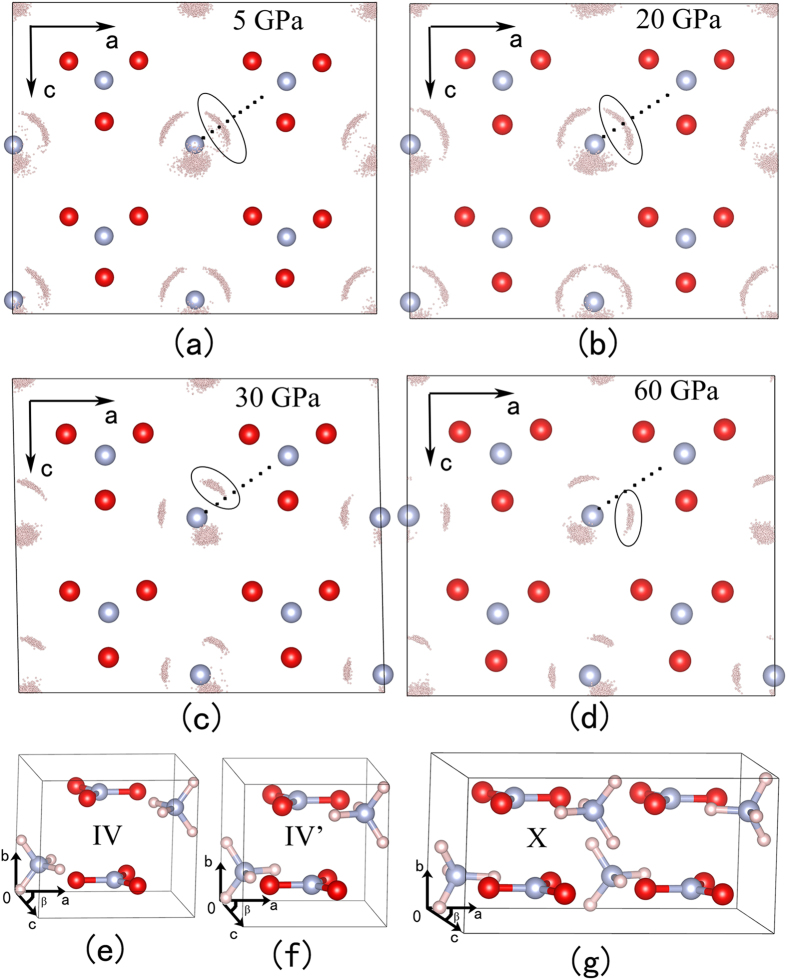
The “H atom cloud” and crystal structures of phase IV, IV’, and X at 300 K. The “H atom cloud” at (**a**) 5 GPa, (**b**) 20 GPa, (**c**) 30 GPa, and (**d**) 60 GPa. The crystal structures of (**e**) AN-IV, (**f**) AN-IV’, and (**g**) AN-X.

For convenience in discussion, the supercell is divided into four layers which are labeled as A, B, C and D layer, as shown in Fig. 1a. The “H atom cloud” in C layer along b-axis are shown in Fig. 3. In Fig. 3a, it is broadest near the middle and tapering toward both ends of the circled “H atom cloud”. Moreover, the H atoms are symmetric about the dotted line between two N atoms. As the pressure increased to 20 GPa (see Fig. 3b), H atoms in the middle of circled “H atom cloud” gradually move to both ends, indicating that the vector of N-H bond points to one O atom more probably than the middle of two O atoms. At 30 and 60 GPa, the circled “H atom cloud” gather closely together and stay away from the dotted line, corresponding to two new structures, as shown in Fig. 3c,d.

**Figure 4:**
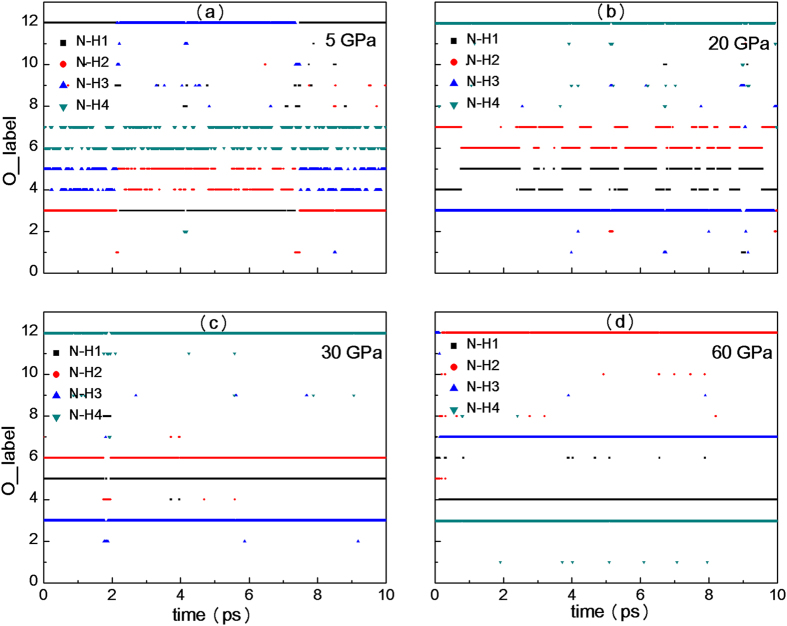
Orientation of ammonium cation as a function of time at 300 K. Orientation of ammonium cation as a function of time at (**a**) 5 GPa, (**b**) 20 GPa, (**c**) 30 GPa and (**d**) 60 GPa. All colors lines are composed of dots got from MD steps.

Much more detailed information about the “H atom cloud” can be explored by examining the relative orientations of four N_H_-H_i_ (i = 1, 2…4) bonds in [NH_4_]^+^ at every MD steps. Without loss of generality, we trace the ammonia cation in the central area of Fig. 3a–d. Considering that O atoms only slightly vibrate near the equilibrium position, we choose 12 O atoms (O_1_, O_2_…O_12_) which neighbor N_H_ as a reference system to show [NH_4_]^+^ orientations, as shown in Fig. 1b. Detailed information on the reference system is presented in the Supporting Information (see Supplementary Figure S1 online). To estimate the direction of N_H_-H_i_ bond, we calculate the degrees of angle N_H_H_i_O_j_ (j = 1, 2…12; see Fig. 1b) and record the label of O atom corresponding to the biggest angle N_H_H_i_O_j_, as shown in Fig. 4. In this scatter diagram, the horizontal axis shows the MD steps and the ordinate axis gives the label of O atoms in Fig. 1b. At a certain time step, the ordinate value of a discrete dot indicates the O atom which the N-H bond points to.

In Fig. 4a, if one makes no distinction among four H atoms in [NH_4_]^+^ and refers to Fig. 3a, then one can see that a N-H bond swings frequently between O_4_ and O_5_; another N-H bond swings frequently between O_6_ and O_7_; two other N-H bonds always point to the neighborhood of O_3_ and O_12_, separately. On the other hand, distinguishing the H atoms by color, for example N-H_1_ bond (black dots), it points to O_12_ at the beginning of simulation, and then points to O_3_ between 2 and 7.3 ps, and then points to O_12_ again in the following simulation time. Combined with other three N-H bond results, it can be seen that the [NH_4_]^+^ undergoes a rotation along N-H_4_ bond even after 5 ps, indicating that the [NH_4_]^+^ in AN can rotate along one N-H bond at 5 GPa. In Fig. 4b, there are also two N-H bonds which change their directions between two O atoms, but the frequency is much lower than that of Fig. 4a. In Fig. 4c, N-H_1_, N-H_2_, N-H_3_ and N-H_4_ bond almost always point to the neighborhood of O_12_, O_4_, O_6_, and O_3_, respectively. Figure 4d exhibits a similar conclusion as Fig. 4c. As seen in Fig. 3 and 4, two N-H bonds which point to O atoms in C layer gradually reduce their reorientation frequency with the increasing of pressure and point to the specific O atoms under enough high pressure.

**Figure 5:**
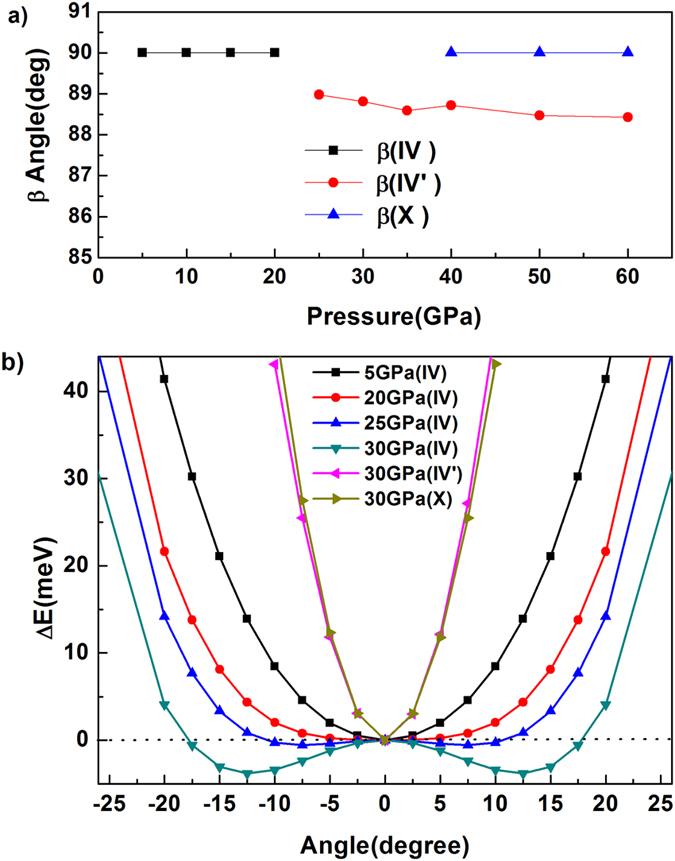
The β angle and total energies of all phases investigated in the present work. (**a**) The *β* angle of AN-IV, AN-IV’ and AN-X as a function of pressure at 300 K. (**b**) The total energies of AN-IV at 5, 20, 25 and 30 GPa, AN-IV’ and AN-X at 30 GPa as a function of the rotational angle. The energies without rotation are scaled as the zero energy.

Three different primitive cells are obtained by making statistical average over the “H atom cloud”, as shown in Fig. 3d–f. The first structure (Fig. 3d) is in agreement with the experimentally proposed AN-IV. The second structure (Fig. 3e), which is considered to be the metastable phase IV’ observed in experiments, has a slightly distorted monoclinic lattice with *P*2_1_/*m* symmetry. It is the first time to uncover the third structure (Fig. 3f), which is defined as AN-X and identified as orthorhombic structure with the space group *Pnma*. The lattice parameters of AN-IV’ and AN-X at 30 and 60 GPa are listed in Table 1, respectively. At the same time, the *β* angles of AN-IV, AN-IV’ and AN-X as a function of pressure at 300 K are shown in Fig. 5a. From this graph, one can see that the *β* angle in AN-IV’ is slightly smaller than 90°, which is different from AN-IV and AN-X. We speculate that the slightly change in *β* angle is the inducement of phase IV-IV’ transition.

To investigate the effect of *β* angle in the phase IV-IV’ transition at 300 K, both the simulations in the *NPT* and *NVT* ensemble are performed at approximately 20 GPa. In the *NPT* ensemble, the structure of AN-IV’ is chosen as the initial structure, and then MD simulation is performed at 20 GPa and 300 K. For most of time, the crystal structures are AN-IV. However, in some time period, when mean value of *β* angles is slightly smaller than 90°, crystal structures transform to AN-IV’. For the *NVT* ensemble, the initial structure is obtained by adjusting the lattice parameters of AN-IV to be the same as AN-IV’ (a = 10.59 Å, b = 9.23 Å, c = 9.05 Å, and *β* = 88.95°) at 20 GPa and keeping the relative positions of atoms in AN-IV invariant. By observing the snapshots during this *NVT* simulation, we find that the crystal structure is the same as AN-IV’. These two simulation results prove that some deviation from 90° in *β* angle indeed plays a crucial role in the formation of AN-IV’, which can explain why phase IV-IV’ transition only appears under conditions of nonhydrostatic pressure.

To investigate the effect of lattice strains in the stabilization of AN-IV’ at 300 K, we perform simulations in the *NVT* ensemble at approximately 50 GPa. Firstly, we optimize the structures of AN-IV’ and AN-X at 0 K and 50 GPa. Then, we keep the relative position of atoms in the structures generated from the first step and exchange the lattice parameters of two phases at 300 K and 50 GPa. By this means, we obtain two kinds of initial structures for MD simulations: one has the relative position of atoms in AN-IV’ at 0 K and the lattice parameters of AN-X at 300 K; another has the consistent relative position of atoms with AN-X at 0 K and the lattice parameters of AN-IV’ at 300 K. Then, we perform MD simulations with above structures in the *NVT* ensemble. We discover that the configuration change from AN-IV’ to AN-X in the first simulation, oppositely, there is few variation in the second simulation. Compare the results between these two simulations: Note that some deviation from 90° in *β* angle plays a crucial role in the stabilization of AN-IV’.

The phase IV-IV’ transition mechanism can be further explored by observing the total energy of AN-IV and AN-IV’ as a function of the rotational angle at different pressures (see Fig. 5b). In the calculations, we keep the position of N_H_ atoms unchanged and gradually rotate ammonium cation along the axis which is parallel to the b coordinate axis. For AN-IV, the potential well is gradually widening with the increase of pressure, and a symmetric double-well potential appears above 20 GPa. The minimum of double-well potential at positive angle area corresponds to the situation that 4 N_H_-H_i_ bonds point to the neighborhood of O_3_, O_5_, O_6_, and O_12_, respectively. The minimum at negative angle area is corresponding to 4 N_H_-H_i_ bonds point to the neighborhood of O_3_, O_4_, O_7_, and O_12_, respectively. Two situations of the reorientation of ammonium cations will occur equiprobably under hydrostatic pressure. One can speculate that slight deviation from 90° in *β* angle will be beneficial in unifying the orientations of ammonium cations in the same layer at high pressure. Then the crystal structure will transform from AN-IV to AN-IV’.

**Figure 6:**
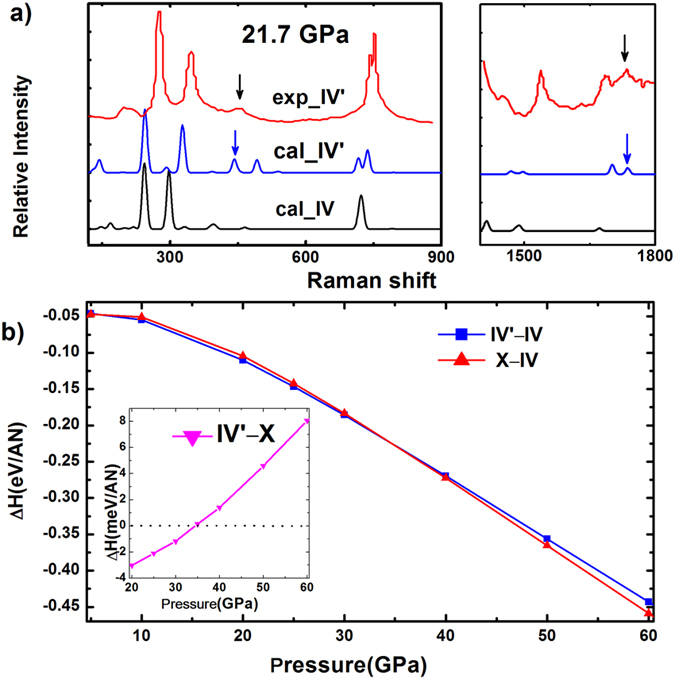
Raman spectra and calculated enthalpies per AN unit as the function of pressure. (**a**) Raman spectra of our simulated AN-IV and AN-IV’ phase compared with experimental results of AN-IV’ at 21.7 GPa. The Raman spectra of experiment, simulated AN-IV’ and AN-IV phase are denoted as red, blue and black lines, respectively. The black arrows show the peaks which signify the phase IV-IV’ transition in experiment. The blue arrows show the simulated peaks correspond to the experiment results. (**b**) Calculated enthalpy difference curves of AN-IV’ and AN-X relative to AN-IV as a function of pressure. The inset: the enthalpy difference of AN-IV’ and AN-X as a function of pressure.

To demonstrate that the structure of AN-IV’ proposed in this work is the metastable phase IV’ found in experiment, we simulate the Raman spectra of AN-IV and AN-IV’ at 21.7 GPa (Fig. 6a). Because the orientations of [NH_4_]^+^ is a major difference between AN-IV and AN-IV’ in our simulations, the Raman peaks involved [NH_4_]^+^ are particularly significant. It is noticed that the ν’_2_([NH_4_]^+^) mode at around 1730 cm^−1^, which is used to confirm the formation of the phase IV’ in the experiment^11^, dose exist in our simulated Raman spectra of AN-IV’ at 21.7 GPa. However, there is no ν’_2_([NH_4_]^+^) mode in our calculated Raman spectra of AN-IV. In addition, the new peak for R([NO_3_]^−^), which is observed in the low frequency region around 400 cm^−1^ by experiment, appears in our calculated Raman spectra of AN-IV’. Highly agreement between the theoretical and experimental results provides a powerful support for the existence of the AN-IV’.

At first glance, the monoclinic structure of AN-IV’ (*P*2_1_/*m*) proposed in the present work is different from the experimentally suggested orthorhombic structure (*Pmmn*). It’s worth noting that the XRD can’t confirm the position of H atoms and the *β* angle of our proposed AN-IV’ is extreme approaching 90° (see Fig. 5a). If the H atoms in our proposed AN-IV’ are eliminated, it has a same symmetry (*Pmmn*) with the experimentally suggested structure. Therefore, it is convinced that the phase AN-IV’ proposed in this study is the metastable phase IV’ observed in experiments.

Geometry optimizations are performed with full structural relaxation including atomic positions and lattice constants at 0 K to obtain the enthalpy difference curves of AN-IV’ and AN-X relative to AN-IV (Fig. 6b). AN-IV’ and AN-X have lower energy than AN-IV, suggesting that AN-IV’ and AN-X are more stable than AN-IV in the pressure range of this study. This result is different from the experimental results that phase IV transformed into phase IV’ at about 20 GPa and room temperature. However, extending the phase boundaries of our MD simulations to low temperature and low pressure (see Supplementary Figure S2 online), one can see that AN-IV’ is stable until 0 GPa and 0 K. This is consistent with the results of calculated enthalpies at 0 K. Therefore, the contradiction between calculated (enthalpies) and measured stable pressure region of phase IV can be attributed to temperature effect. The inset in Fig. 6b displays the enthalpy difference of AN-IV’ and AN-X at 0 K, showing that AN-X is more stable than AN-IV’ above 35 GPa.

**Figure 7:**
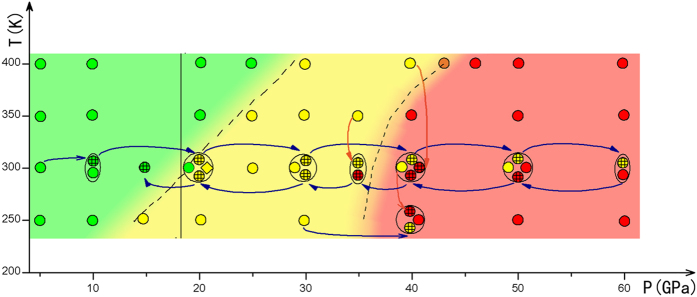
Phase diagram and simulation protocol of AN. Small circles in one big circle or ellipse have a same target pressure and temperature. Small circles with cross lines: the result at the beginning of arrow condition as the initial structure of the end of arrow condition. Small circles without cross lines: the optimized AN-IV at relevant pressure and temperature as initial structure. Rhombus: *NVT* ensemble. Green circles represent the structure of AN-IV. Yellow circles represent the structure of AN-IV’. Red circles represent the structure of AN-X. Orange circles (43 GPa and 400 K): There are mutual transformation between AN-IV’ and AN-X in the simulation. Orange arrow: simulated annealing process. The vertical line at about 18 GPa is the IV’-IV phase boundary given in experiment^21^.

The simulation protocol, together with majority of the simulation results for AN, is schematically shown on Fig. 7. At 300 K, phase IV-IV’ transition pressure is approximately 20 GPa, which is almost identical to the experimentally observed 18 GPa. During MD simulation at 43 GPa and 400 K, the mutual transformation between AN-IV’ and AN-X are observed, suggesting that 43 GPa are very close to the boundary of phase IV′-X transition at 400 K. There are also yellow spots (AN-IV′) in the red area (AN-X), which can be explained by Fig. 5b. In this graph, it is obvious that the total energies of AN-IV’ and AN-X increase sharply with the change of angle at 30 GPa, indicating that the spatial orientation of ammonium cation will be strongly restrained when the pressure is over 30 GPa. Thus, once the AN-IV’ formed, it is difficult to transform into AN-X at low temperature.

## Conclusion

We explore the high-pressure phase transition of ammonium nitrate through ab initio molecular dynamics simulations. The existence of phase IV’ is confirmed and a new phase, AN-X, is discovered. In addition, through the *NVT* ensemble simulations, we reveal that lattice strains play an important role in formation and stabilization of phase IV’. The above fact can explain the proposed conclusion in experiment that phase IV-IV’ transition is induced by shear stress under nonhydrostatic pressure. Finally, the phase diagram of AN is determined at pressure range of 5–60 GPa and temperature range of 250–400 K.

## Methods

In this study, we use *ab initio* molecular dynamics simulations based on density functional theory implemented in the Vienna *ab initio* simulation package (VASP) code^27^,^28^,^29^ to schematically investigate AN. The projector-augmented wave (PAW) method^30^,^31^ is adopted and exchange-correlation functions are treated within the Perdew-Burke-Ernzerhof generalized gradient approximation (GGA)^32^. A plane-wave basis-set cutoff energy of 520 eV and 2 × 2 × 2 Monkhorst−Pack k-point mesh^33^ are employed. Simulations are implemented both in the *NPT* (*N*-constant number of particles, *P*-constant pressure, and *T*-constant Temperature) ensemble^34^ and *NVT* (*V*-constant volume) ensemble^35^,^36^,^37^ for a system containing 16 AN molecules.

Considering the computational efficiency and the feature of MD, the simulations are performed in two ways. First, we optimize the supercell of AN-IV (Fig. 1) at 0 K and a series of pressures (5, 20, 40 and 60 GPa) as initial structures. We heat the structures at a series of temperatures (250, 300, 350 and 400 K) using their corresponding pressures. All the simulation conditions and results using this method are shown by small circles without cross lines in Fig. 7. Second, we choose the results of finished simulations as initial structures and perform MD simulations at new conditions. For example, at temperature of 300 K, we use the result of 5 GPa as initial structure for the simulation at 10 GPa, then we gradually increase pressure in 10 GPa steps until 60 GPa. In a similar manner, we use the result of 60 GPa as initial structure and decrease pressure to 15 GPa in the interval pressure of 10 or 5 GPa at 300 K. The whole simulation process of increasing and decreasing pressure is shown on Fig. 7 and marked as blue arrows. In addition, we also show the annealing process by the orange arrows, which are signed by small circles with cross lines. In addition, we employ simulation times of 20–30 ps (with time step of 1 fs) at the vicinity of the phase boundary, and at least 10 ps at the other conditions.

We employ density functional perturbation theory calculations with CASTEP (Cambridge Serial Total Energy Package)^38^ code to study the Raman spectra of AN-IV and AN-IV’ at 21.7 GPa. GGA-PBE for the exchange-correlation functional, norm-conserving potentials, the cutoff energy of 750 eV and the Monkhorst-Pack k-points of 5 × 6 × 5 for corresponding Brillouin zone have been applied.

## Additional Information

**How to cite this article**: Yu, H. *et al.* *Ab initio* molecular dynamic study of solid-state transitions of ammonium nitrate. *Sci. Rep.* **6**, 18918; doi: 10.1038/srep18918 (2016).

## Supplementary Material

Supplementary Information

## Figures and Tables

**Table 1:** Lattice constants and atomic coordinates at 300 K for AN-IV’ at 30 GPa, and AN-X at 60 GPa.

Phase	Structure	Parameters (Å, deg)	Atom	*x*	*y*	*z*
AN-IV’	*P*2_1_/*m*	*a* = 5.1486	H1 (4f)	0.78565	0.93601	0.01331
		*b* = 4.4716	H2 (2e)	0.46161	0.25000	0.13796
		*c* = 4.4203	H3 (2e)	0.83446	0.75000	0.68607
		*β* = 88.79	N1 (2e)	0.25484	0.25000	0.11423
			N2 (2e)	0.25041	0.75000	0.48957
			O1 (2e)	0.03931	0.75000	0.35393
			O2 (2e)	0.46085	0.75000	0.34953
			O3 (2e)	0.24448	0.75000	0.77631
AN-X	*Pnma*	*a* = 8.4855	H1 (8d)	0.99629	0.55463	0.81031
		*b* = 4.1937	H2 (4c)	0.56297	0.25000	0.03352
		*c* = 4.8746	H3 (4c)	0.82459	0.75000	0.81541
			N1 (4c)	0.56282	0.25000	0.24322
			N2 (4c)	0.74310	0.75000	0.24136
			O1 (4c)	0.67467	0.75000	0.46447
			O2 (4c)	0.66829	0.75000	0.02289
			O3 (4c)	0.89065	0.75000	0.23245
