# Supplementary material for: Ab initio molecular dynamic study of solid-state transitions of ammonium nitrate
Source: Sci Rep. 2016 Jan 12;6:18918. doi: 10.1038/srep18918 (PMC4709593; doi:10.1038/srep18918)

**Supplementary information**

Ab initio molecular dynamic study of solid-state transitions of ammonium nitrate

Hongyu Yu^1^, Defang Duan^1^, Hanyu Liu^2^, Ting Yang^1^, Fubo Tian^1^, Kuo Bao^1^, Da Li^1^, Zhonglong Zhao^1^, Bingbing Liu^1^ & Tian Cui^1^

^1^State key Laboratory of Superhard Materials, College of Physics, Jilin University, Changchun, 130012, P. R. China, ^2^Department of Physics and Engineering Physics, University of Saskatchewan, Saskatoon, S7N 5E2, Canada

**Figure S1** | reference systems.


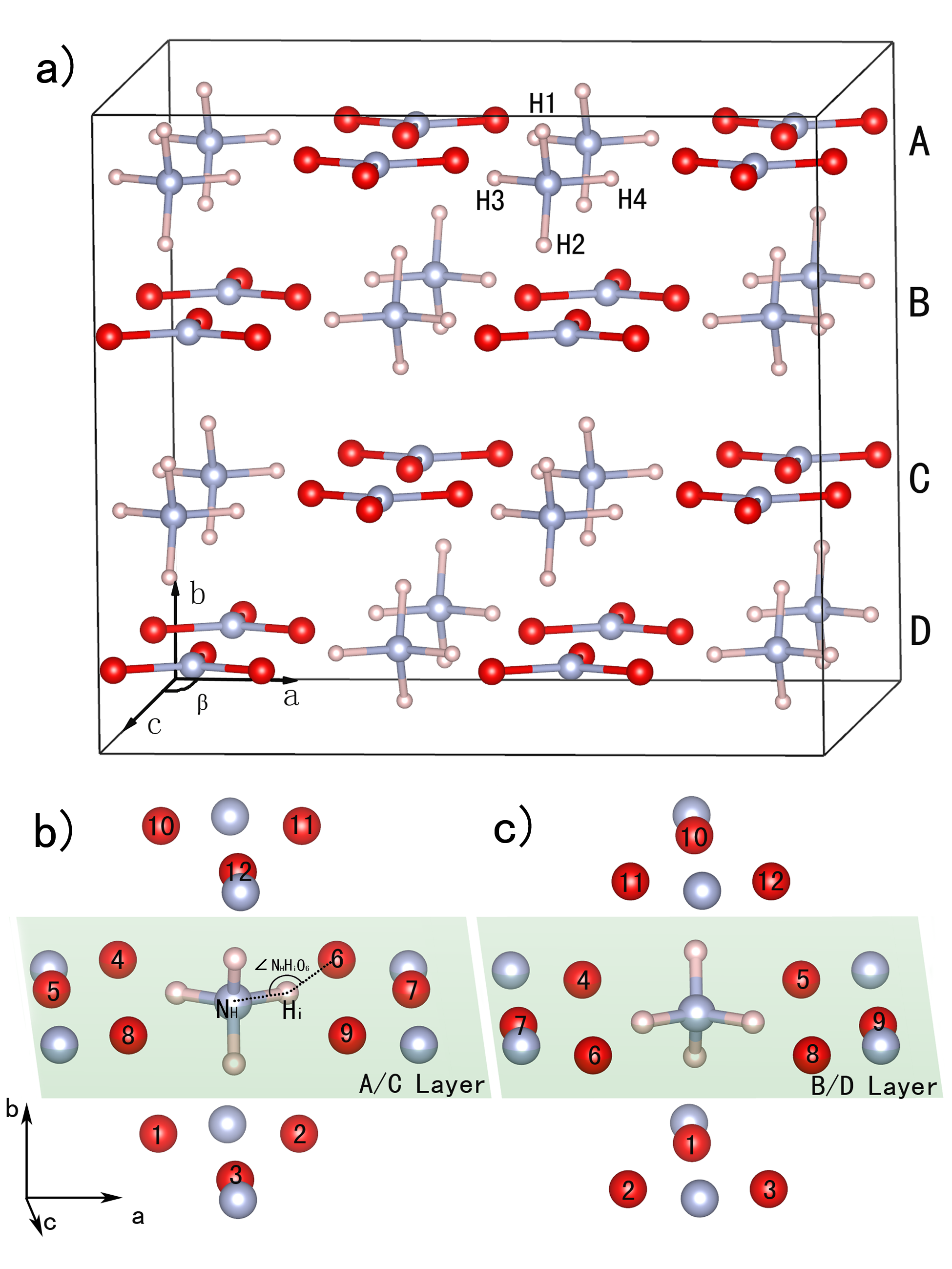


There are 16 ammonium cations in the supercell (see Figure S1a), meaning that there are should be 16 reference systems to explore the informations about the “H atom cloud”. Considering periodic boundary conditions and symmetries, all reference systems can be summarized into two categories: A and C layers in Figure S1b, B and D layers in Figure S1c. Without loss of generality, we adopt the reference system shown in Figure S1b to show the orientation of [NH_4_]^+^ at every MD step by describing the direction of four N_H_-H_i_ bonds in Figure S1b.

**Figure S2** | The extended phase boundaries of AN.


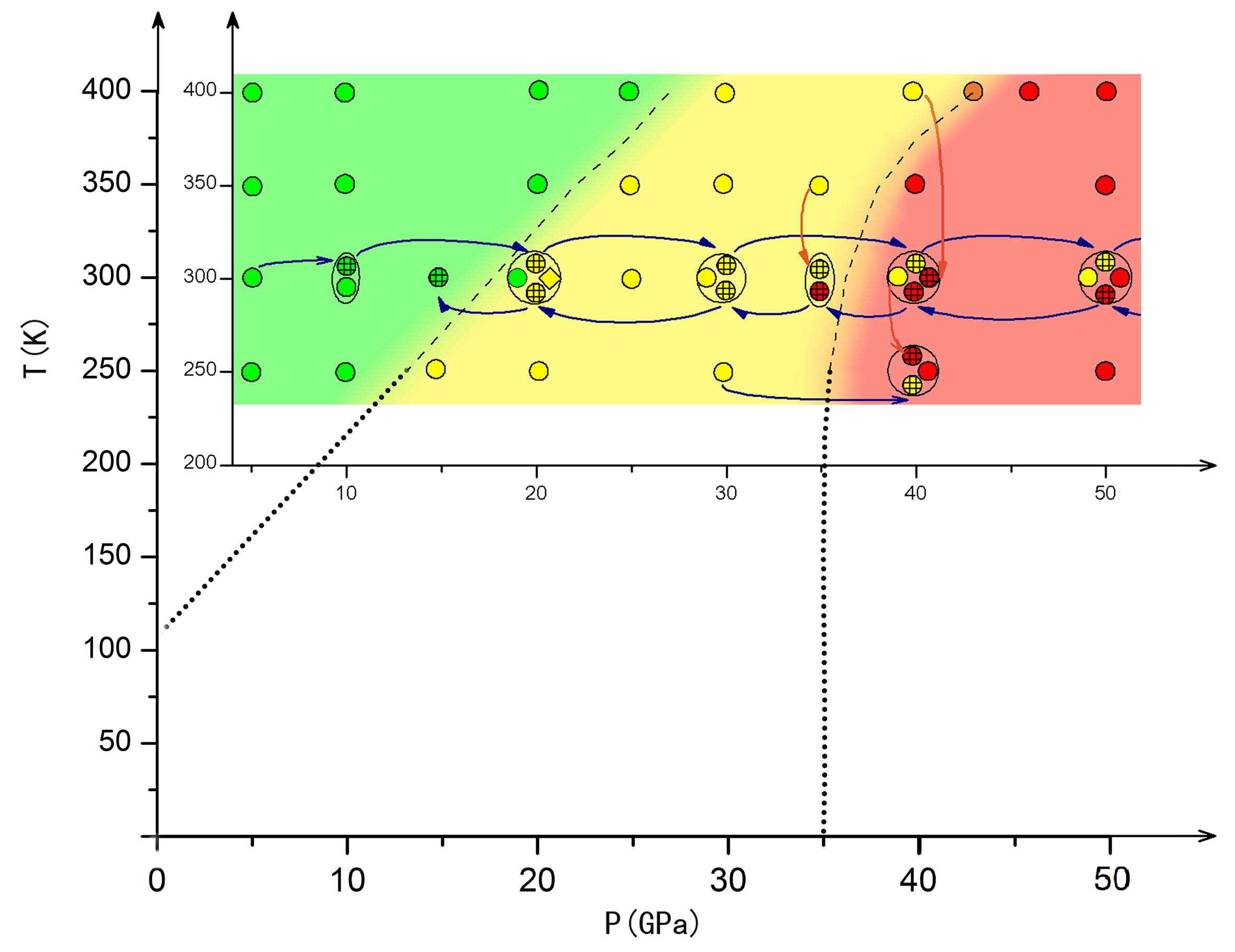

Supplement: Supplementary Information [file srep18918-s1.doc]
